# Familial Juvenile Polyposis Syndrome: A Case Report of a Cosegregation of SMAD4 Germline Pathogenic Variant c.403C>T and Review of the Literature

**DOI:** 10.1155/cris/8105440

**Published:** 2026-08-30

**Authors:** Carlos Augusto Real Martinez, Giovanna Savoy Pazin, Rayama Moreira Siqueira, Rita Barboza de Carvalho, Michel Gardere Camargo, Lívia Moreira Genaro, Pedro Henrique Leite Bonfitto, Maria de Lourdes Setsuko Ayrizono, Raquel Franco Leal, Cláudio Saddy Rodrigues Coy

**Affiliations:** ^1^ Colorectal Surgery Unit, Department of Surgery, Universidade Estadual de Campinas (UNICAMP), Campinas, São Paulo, Brazil, unicamp.br; ^2^ Colorectal Division, School of Medicine, São Francisco University (USF), Bragança Paulista, São Paulo, Brazil, usf.edu.br; ^3^ Post-graduate Program in Surgical Sciences, School of Medical Sciences, University of Campinas (Unicamp), Campinas, São Paulo, Brazil, unicamp.br; ^4^ Pathology Division, Gastrocenter, University of Campinas (Unicamp), Campinas, São Paulo, Brazil, unicamp.br

**Keywords:** case report, colorectal neoplasms, intestinal polyposis, juvenile polyposis syndrome, SMAD4 protein

## Abstract

Juvenile polyposis syndrome (JPS) is an inherited autosomal dominant disease that is distinguished by the emergence of numerous juvenile polyps within the gastrointestinal (GI) tract and has an increased risk of cancer. JPS is associated with germline mutations of the *BMPR1A* or *SMAD4* genes. We aim to describe a case of familial occurrence of the SMAD4 germline pathogenic variant c.403C>T in a Brazilian family. We present a case of an 18‐year‐old female with rectal bleeding and chronic anemia who had a family history of colorectal issues—her mother had a total colectomy due to multiple polyps, and her maternal grandfather died from colorectal cancer (CRC). Despite a negative physical examination for attenuated familial adenomatous polyposis (AFAP) signs, a colonoscopy revealed around 30 proximal colon polyps of juvenile nature. These were systematically removed through multiple sessions, and an upper digestive endoscopy uncovered a few sessile inflammatory polyps. Notably, no polyps were found in the small intestine via double‐balloon enteroscopy. After polyp removal, her bleeding episodes ceased, and her anemia resolved. She now maintains her health through biennial colonoscopies, upper digestive endoscopies, and endoscopic capsule procedures. Genetic testing identified a pathogenic mutation in exon 3 of the SMAD4 gene (c.403C>T), resulting in a truncated SMAD4 protein (p.Arg135 ^∗^). While previously documented in international databases, this represents the first report of a Brazilian family carrying this variant. Additionally, no mutations were found in the BMPR1A gene during genetic testing. In conclusion, molecular analysis confirmed the familial occurrence of the SMAD4 germline pathogenic variant c.403C>T within this Brazilian family.

## 1. Introduction

Hamartomatous polyposis syndromes (HPS) comprise a heterogeneous group of inherited disorders characterized by the development of hamartomatous polyps (HPs) throughout the gastrointestinal (GI) tract. This group includes juvenile polyposis syndrome (JPS), Peutz–Jeghers syndrome (PJS), and PTEN‐associated hamartoma tumor syndrome (PHTS). Despite exhibiting unique clinical presentations, histological discrimination between the HPs characteristic of these syndromes poses significant difficulties [1]. JPS (OMIM 174900; MedGen UID: 87518) is a rare autosomal dominant HPS distinguished by the emergence of numerous juvenile polyps within the GI tract [1–6]. Individuals with JPS face a substantially increased lifetime risk of colorectal cancer (CRC), together with elevated susceptibility to additional GI malignancies. Because these syndromes predispose patients to GI cancer, timely recognition and diagnosis are essential for appropriate surveillance and management [2].

The diagnosis of JPS is established based on specific clinical criteria and confirmed by molecular genetic testing, independent of other hamartomatous syndromes [4, 7]. The inheritance pattern is autosomal dominant, and most patients with JPS develop colorectal HPs by the age of 20 years [1–4]. The consideration of JPS should arise when evaluating a proband presenting with the subsequent clinical and histopathologic attributes: (1) the manifestation of anemia, rectal bleeding, and rectal polyp prolapse; (2) more than one juvenile polyp; and (3) one or more juvenile polyps concomitant with a documented family history of JPS.

Advances in molecular genetics have demonstrated that pathogenic germline variants involving SMAD4, BMPR1A, PTEN, and ENG contribute to the development of juvenile polyps in genetically predisposed individuals. Most molecularly confirmed cases of JPS are associated with pathogenic germline variants affecting either BMPR1A or SMAD4 [1–9]. Therefore, genetic testing for mutations in these genes is imperative for establishing a molecular diagnosis in clinically suspected cases of JPS. Germline pathogenic variants for *SMAD4* or *BMPR1A* are detected in ~40%–60% of probands meeting established clinical diagnostic criteria for JPS [4, 9]. The diagnosis of JPS is primarily established through recognized clinical criteria: (1) five or more juvenile polyps in the colon or rectum; (2) juvenile polyps distributed throughout the GI tract; or (3) any number of juvenile polyps in individuals with a documented family history of JPS. While these clinical features provide the primary diagnostic framework, molecular genetic testing for pathogenic variants in SMAD4 or BMPR1A serves as the gold standard for definitive confirmation and is crucial for establishing a molecular diagnosis in clinically suspected cases [9, 10].

The present study aims to present a case of a patient with JPS whose genetic test identified a previously described heterozygous pathogenic variant in exon 3 of the SMAD4 gene (c.403C>T).

## 2. Case Presentation

**Figure 1:**
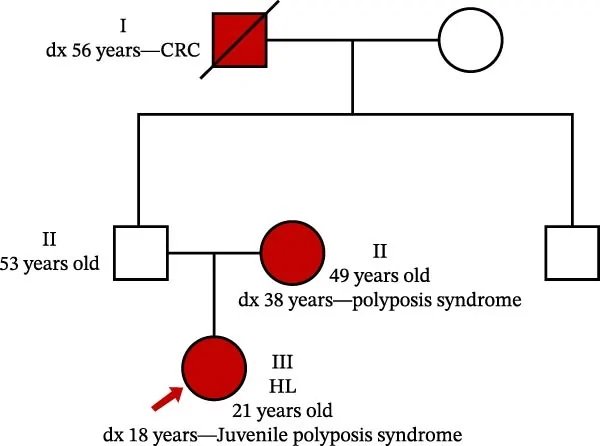
Heredogram of the family. The heredogram illustrates the familial occurrence of the SMAD4 pathogenic variant in the proband (III) and her mother (II‐2). In this diagram, an arrow points to the specific individual who is the subject of this report, signifying the patient under investigation. The maternal grandfather (I) died of colorectal cancer at age 56; however, genetic testing was only possible for individuals II and III due to the unavailability of biological samples from the first generation (II‐1, 53 years old).

An 18‐year‐old female has complained of abdominal colic, chronic anemia, and rectal bleeding with bowel movements since she was 6 years old, mainly associated with episodes of intestinal constipation. The patient reported that her mother had undergone total colectomy with ileorectal anastomosis in another hospital 20 years prior due to a diagnosis of attenuated familial adenomatous polyposis (AFAP). The proband’s father was unavailable for clinical examination or genetic testing. Consequently, molecular analysis was conducted only on the available affected family members (the proband and her mother). She also reported that her maternal grandfather died of CRC at age 56 (Figure 1). While the maternal grandfather’s clinical history is highly suggestive of the syndrome’s progression in the family, the lack of available genetic material due to his death precludes a formal three‐generation segregation analysis. Therefore, the presence of the SMAD4 variant in the proband and her mother is considered a familial occurrence. The patient did not have any other extraintestinal manifestations related to AFAP.

**Figure 2:**
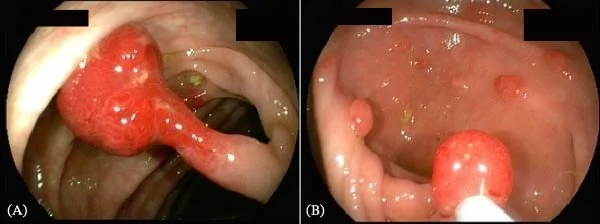
Colonoscopy findings. (A) A pedunculated polyp is observed in the right colon. (B) Sessile polyps in the transverse colon.

With clinical suspicion that the patient had AFAP, she was referred for a colonoscopy. The colonoscopy identified the presence of ~30 rounded sessile and pedunculated polyps with diameters ranging from 3 to 30 mm, which affected the entire colon, especially the cecum and ascending colon (Figure 2A,B). No polyps were identified in the rectum, and all identified polyps were removed in three scheduled colonoscopies. Upper digestive endoscopy also identified several sessile polyps, and double‐balloon enteroscopy did not identify polyps in the small bowel. A brain magnetic resonance imaging (MRI) scan did not reveal the presence of arteriovenous malformations.

**Figure 3:**
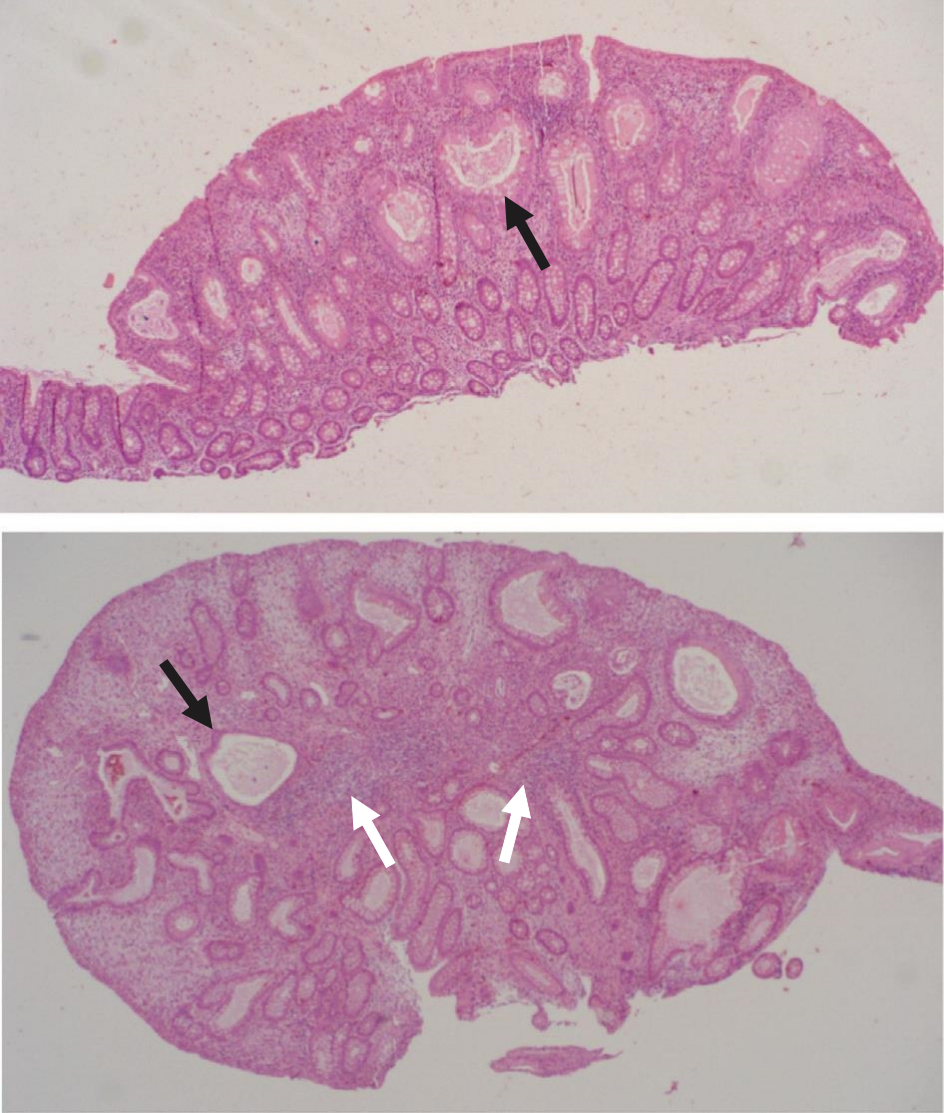
Histopathological findings. Histopathological examination of colon tissues reveals juvenile polyps (HE‐25x magnification). The colonic mucosa displays notable characteristics, including dilated crypts (indicated by the black arrow), which occasionally appear irregular. Additionally, the lamina propria is marked by an abundance of inflammatory infiltrate (highlighted by the white arrow). HE, hematoxylin–eosin.

The histopathological study was conducted by expert GI pathologists from our institution. Analysis of the resected colonic polyps showed that they had the histological characteristics of juvenile polyps (Figure 3). The epithelium appeared normal, without areas of dysplasia, and the lamina propria was abundant, containing elongated, dilated, and mucus‐filled cystic glands with an associated chronic inflammatory infiltrate in the stroma, which are pathognomonic hallmarks of juvenile polyps. In all removed polyps of the stomach, histopathological studies showed that they were inflammatory or hyperplastic polyps. When these Morson criteria are fully met, the morphology alone is sufficient to distinguish JPS from other HPS, such as Peutz–Jeghers or Cowden syndrome.

Due to the hamartomatous histology of colon polyps (juvenile polyps) associated with the previous unconfirmed diagnosis of AFAP in her mother, we have chosen to perform a genetic panel (INVITAE; Invitae Diagnostic Testing Results, Invitae 1400 16th Street, San Francisco, CA 94103, USA), which is a CLIA‐certified and CAP‐accredited facility. The analysis used next‐generation sequencing (NGS) to evaluate 84 genes most commonly involved in hereditary CRC syndromes, including full gene sequencing and copy number variation (CNV) analysis (deletions and duplications). The laboratory achieves a median depth of coverage of at least 50x, with more than 99% of the targeted bases covered at a depth of greater than 20x. The genetic panel identified a mutation in exon 3 of the SMAD4 gene (c.403C>T) that caused the transcription of a truncated SMAD4 protein (p.Arg135 ^∗^).

The identification of this variant in this patient and her mother confirms the familial presence in a Brazilian family, correcting a previous clinical misdiagnosis of AFAP. This premature translational stop signal has been observed in individuals with JPS. The identified variant had been described previously (PMID 33097490, 22810475, 17362581, 16152648, and 16436638) [11–15]. ClinVar contains an entry for this variant (Variation ID: 24802). According to ACMG/AMP guidelines, this variant was classified as pathogenic based on the following evidence: PVS1 (null variant in a gene where LOF is a known mechanism), PM2 (absent from population databases like gnomAD), and PP5 (previously reported as pathogenic by reputable sources such as ClinVar). This variant meets the requisite criteria for pathogenicity based on functional and clinical evidence. The same genetic panel was applied for the patient’s mother, and she carries the same mutation. Thus, the previously considered diagnosis of AFAP was ruled out.

Currently, the patient in this report undergoes surveillance with upper digestive endoscopy and colonoscopy every 1–2 years, a small bowel examination with an endoscopic capsule every 2–3 years, and biennial abdominal CT enterography.

## 3. Discussion

HPS is a group of hereditary diseases that includes HPs of the digestive tract as one of their main features [14–16]. HPS encompasses a group of genetic disorders that are rarely described, including PJS, JPS, and PTEN hamartoma tumor syndromes (Bannayan–Riley–Ruvalcaba and Cowden syndrome) [1, 12]. While HPs of the colorectum predominantly occur within the context of inherited polyposis syndromes, they may also manifest as sporadic lesions [16].

JPS is an uncommon inherited disorder, with an estimated prevalence ranging from ~1 per 100,000 to 1.6 per 160,000 individuals [1, 5, 6], being a rare autosomal dominant disorder characterized by incomplete penetrance and variable expressivity. Notably, only 20%–50% of affected individuals report a positive family history, reflecting a significant proportion of de novo pathogenic variants [2]. JPS exhibits multiple juvenile GI polyps, with 98% displaying colonic or rectal involvement, compared to stomach (14%), duodenum (7%), and small bowel (7%) presentations [1, 4, 17–19]. Most colonic polyps (~70%) occur in the proximal colon [7]. JPS patients are usually asymptomatic, but when they develop symptoms, rectal bleeding with concomitant anemia, as occurred in the patient in this report, is the most common presentation [1]. Recurrent abdominal colic, diarrhea, dysentery, and eventually intestinal intussusception can also occur at the sites of the largest polyps [1, 4, 7, 19]. Patients with JPS have a high risk of CRC, ranging from 39% to 68% [1, 20].

The diagnosis of JPS is based on well‐recognized clinical criteria, including the presence of multiple juvenile colorectal polyps, juvenile polyps involving different regions of the GI tract, or any juvenile polyp occurring in an individual with a confirmed pathogenic JPS‐associated variant [4, 19]. A subset of patients exhibits extraintestinal manifestations, including cutaneous findings (e.g., telangiectasia and pigmented nevi) and skeletal stigmata (macrocephaly, hydrocephalus, cleft palate, polydactyly, and hypertelorism), reported prevalences of 56% and up to 70% for cutaneous and skeletal phenotypes, respectively [4, 21].

It is essential to highlight that “juvenile polyps” denote a specific histopathological subtype characterized by cystically dilated glands within an expanded lamina propria rather than implying a pediatric onset. Clinicians should note that this terminology may inadvertently suggest an early disease presentation, potentially confounding differentiation from AFAP. The clinical overlap between JPS and AFAP led to a 20‐year diagnostic error in the proband’s mother, who was previously treated with AFAP. This case demonstrates that molecular testing is essential for a correct differential diagnosis, preventing inappropriate surgical strategies, and guiding the correct surveillance protocol for HPS. Most JPS patients develop colorectal polyps by the age of 20 years [22]. However, expressivity varies significantly: some mutation carriers exhibit fewer than 10 lifetime polyps, while others develop profuse polyposis (more than 100), resembling aggressive phenotypes [20]. Morphologically, these polyps range from diminutive sessile lesions to large pedunculated masses exceeding 3 cm in diameter [4, 16, 20]. Macroscopically, they typically demonstrate smooth surfaces, are erythematous, and lack fissures or lobules. Large polyps often become multilobulated, with a superficial mucinous exudate and fibrin deposition [4]. The clinical presentation of the current case exhibited all these morphological features.

It is estimated that more than 30% of probands with JPS have no suspicious history of polyposis and have therefore developed a de novo pathogenic mutation. However, ~70% of the remaining individuals with JPS have a positive family history [4, 5]. As the syndrome has a dominant inheritance pattern, according to the classic pattern of transmission, each child of an affected individual has a 50% probability of inheriting the pathogenic mutation.

The genetic basis of JPS lies in germline mutations in either *BMPR1A* or *SMAD4*, which cause changes in the cell signaling pathways of the corresponding proteins. The *SMAD4* gene, located on chromosome 18q21.1, encodes a cytoplasmic effector protein that transduces signals for the TGF‐β superfamily. This pathway regulates critical biological processes, including cellular proliferation, differentiation, and immune regulation [23]. Germline mutations in SMAD4, as documented in the current literature, predominantly comprise minor insertions/deletions and single‐nucleotide variants, resulting in nonsense, splice site, or missense alterations [3, 20, 23, 24]. The most frequently reported SMAD4 germline mutation is a recurrent 4‐base deletion in exon 9, establishing this locus as a recognized mutational hotspot [25, 26].

In the present case, molecular analysis revealed a heterozygous pathogenic variant in exon 3 of the *SMAD4* gene (c.403C>T), resulting from a cytosine‐to‐thymine substitution at codon 403. This nonsense variant creates a premature termination codon (p.Arg135 ^∗^) and is predicted to trigger nonsense‐mediated mRNA decay or produce a truncated protein. Such loss‐of‐function mechanisms are established drivers of JPS [14]. This specific variant (p.Arg135 ^∗^) has been previously documented in JPS patients [14, 15] and is absent from population frequency databases (gnomAD no frequency), although it is cataloged in ClinVar (Variation ID: 24802) as pathogenic. Critically, SMAD4 mutation carriers typically exhibit more aggressive phenotypes compared to those with BMPR1A variants. This includes a higher polyp burden and a significantly increased risk of upper GI polyposis and gastric malignancy. While capsule endoscopy is the primary and preferred surveillance modality for monitoring the small bowel in SMAD4 carriers, alternative tools such as MRI/CT enterography or enteroscopy may be considered for deep initial investigation or therapeutic intervention [21, 22].

Germline mutation analysis in PJS delineates two distinct phenotype profiles: patients harboring *SMAD4* pathogenic variants typically manifest both GI polyps (colonic and gastric) with concomitant hereditary hemorrhagic telangiectasia (HHT), whereas those with *BMPR1A*‐ mutations primarily exhibit a colorectal‐predominant polyposis phenotype [4, 9, 27]. Wain et al. [27] in a retrospective evaluation of 34 patients with a pathogenic mutation of the SMAD4 gene showed that 97% of the affected individuals presented with colonic polyps, more than 68% presented with gastric polyps, and over 70% of these patients exhibited symptoms of HHT. Another study demonstrated that extraintestinal features could occur in up to 30% of cases [22]. Notably, despite carrying the SMAD4 mutation, neither the proband nor her mother displayed clinical signs of HHT, such as epistaxis or telangiectasias. This highlights the variable expressivity of the syndrome; as discussed by Gallione et al. [28], HHT manifestations can be incomplete or delayed in some SMAD4 carriers, necessitating ongoing monitoring for visceral arteriovenous malformations regardless of the initial presentation. A retrospective European multicenter study identified a significantly higher frequency of small intestine polyps in *SMAD4* carriers (15.7%) compared to other genotypes. While capsule endoscopy demonstrates limited sensitivity for quantifying small bowel polyp burden in JPS, alternative modalities, including MRI/CT enterography or enteroscopy, should be considered for comprehensive surveillance in high‐risk individuals [21, 22]. The high risk of developing GI tract cancer in patients with JPS justifies and imposes surveillance strategies to reduce the cancer risk. Thus, periodic colonoscopy, esophagogastroduodenoscopy, capsule endoscopy, or MRI/CT enterography should be offered to patients with JPS [21, 22, 29]. Current guidelines from the American Multi‐Society Task Force on CRC recommend genetic evaluation for individuals meeting any of the following criteria: five or more colorectal juvenile polyps, with two or more juvenile polyps in extracolorectal GI locations, or with any number of juvenile polyps and one or more first‐degree relatives diagnosed with JPS (strong recommendation, low quality of evidence) [29]. Annual colonoscopy is recommended starting at ages 12–15 years (or earlier with symptomatic presentation, particularly hematochezia) with resection of all polyps ≥5 mm (weak recommendation, low quality of evidence) [29]. For small bowel evaluation distal to the duodenum, periodic surveillance via enteroscopy, capsule endoscopy, and/or CT enterography is warranted when duodenal polyposis is present, unexplained iron deficiency anemia or hypoalbuminemia occurs, or symptoms suggest small bowel pathology [29].

Endoscopic polypectomy constitutes first‐line management for patients with oligopolyposis in any GI segment [3]. Colectomy with ileorectal anastomosis is indicated if cancer, high‐grade dysplasia, or polyposis cannot be adequately controlled endoscopically [12]. Surveillance of the remaining rectum or the eventual pouch after an ileoanal pouch anastomosis is necessary [30]. Proctocolectomy with IPAA represents a definitive surgical option when extensive rectal polyposis precludes endoscopic management [12]. Furthermore, ~50% of patients undergoing ileorectal anastomosis subsequently require completion proctectomy due to recurrent rectal polyposis [30]. Complete or partial gastrectomy may also be necessary for patients with advanced dysplasia, gastric cancer, or even massive gastric polyposis that cannot be effectively controlled endoscopically [12].

SMAD4 pathogenic variants exhibit incomplete penetrance in HHT, with clinical manifestations occurring in 15%–81% of carriers. This variable expressivity necessitates vigilant monitoring for HHT‐associated features, including epistaxis, occult GI bleeding, digital clubbing, visceral arteriovenous malformations, and mucocutaneous telangiectasias, all of which occur in individuals harboring SMAD4 mutations [29]. The American Multi‐Society Task Force on CRC guidelines recommend comprehensive HHT surveillance and management for this genetic subpopulation. Specifically, a systemic clinical evaluation for HHT is recommended at the time of initial diagnosis, including screening for pulmonary and cerebral arteriovenous malformations, with appropriate intervention (weak recommendation and low‐quality evidence) [29]. Table 1 summarizes the standardized recommendations for endoscopic and systemic surveillance in JPS.

**Table 1:** Recommendations for the endoscopic surveillance in JPS [26].

GI tract	Cancer risk	Recommendation surveillance	Note
Colon and rectum	39%–68%	Colonoscopy every 1–3 years, depending on the polyps’ burden	Colorectal polyps >5 mm should be removed
Stomach	21%	EGD every 1–3 years	Multidisciplinary management advisable
Small bowel	Undefined	Recommended for symptomatic JPS patients	Capsule endoscopy CT or MRI enterography
Arteriovenous malformations	—	Recommended for JPS patients with *SMAD4* mutations (at diagnosis)	MRI

Abbreviations: CT, computed‐tomography; EGD, esophagogastroduodenoscopy; GI, gastrointestinal; JPS, juvenile polyposis syndrome; MRI, magnetic resonance imaging.

Given the SMAD4 genotype, this family requires rigorous surveillance not only of the colon but also of the stomach due to the high risk of gastric cancer. Furthermore, identifying the specific c.403C>T variant allows for cascade testing of at‐risk relatives, ensuring that noncarriers can be exempted from invasive and costly screening protocols. With this tailored strategy, there is a reduction in health system costs and in the need for frequent invasive endoscopic exams, which are associated with potential complications. Another critical aspect of realizing the indications of genetic tests is the strategies for genetic counseling that should be offered to all individuals who carry the mutation. The surveillance strategy adopted for the proband in this report follows the principles established by these international guidelines and is tailored to the specific risks associated with her SMAD4 pathogenic variant [31]. This approach underscores the importance of integrating formal guidelines with individualized patient management in rare polyposis syndromes. This article presents the first case of a Brazilian family showing the familial occurrence of the SMAD4 germline pathogenic variant c.403C>T. This report underscores the importance of genetic testing in Latin American cohorts to provide accurate diagnosis and cancer surveillance. Careful family history, genetic panel tests, and thorough screening protocols are necessary to accurately identify patients and relatives at risk for JPS. These approaches, associated with regular surveillance, are the most valuable tools for precisely diagnosing JPS and reducing the risk of cancer development.

## Funding

This research did not receive any specific grant from funding agencies in the public, commercial, or not‐for‐profit sectors.

## Ethics Statement

The case report was conducted following the Research Ethics Committee of the University of Campinas, with Approval Number 62849222.7.0000.5404.

## Consent

Informed written consent was obtained from the patient for the publication of this case report and accompanying images.

## Conflicts of Interest

The authors declare no conflicts of interest.

## Data Availability

Data sharing is not applicable to this article, as no new data were created or analyzed in this study.
